# Supplementary material for: Structural Elucidation of an Atropisomeric Entcassiflavan-(4β→8)-Epicatechin Isolated from Dalbergia monetaria L.f. Based on NMR and ECD Calculations in Comparison to Experimental Data
Source: Molecules. 2022 Apr 13;27(8):2512. doi: 10.3390/molecules27082512 (PMC9028727; doi:10.3390/molecules27082512)
Supplement: Supplementary file 1 [file molecules-27-02512-s001.zip › molecules-1602333-supplementary.pdf]

Supporting Information

Structural Elucidation of an Atropisomeric Entcassiflavan-(4β→8)-Epicatechin Isolated from *Dalbergia monetaria* L.f. Based on NMR and ECD Calculations in Comparison to Experimental Data

Patrícia Homobono Brito de Moura [1,2], Wolfgang Brandt [3], Andrea Porzel [3], Roberto Carlos Campos Martins [1], Ivana Correa Ramos Leal [2,*] and Ludger A. Wessjohann [3,*]

[1] Natural Products Research Institute (IPPN), Center of Health Sciences, Federal University of Rio de Janeiro (UFRJ), Rio de Janeiro 21941-902, RJ, Brazil; patricia.homobono@gmail.com (P.H.B.d.M.); roberto.rcc@gmail.com (R.C.C.M.)

[2] Natural Products and Food Department, Pharmacy Faculty, Center of Health Sciences, Federal University of Rio de Janeiro (UFRJ), Rio de Janeiro 21941-902, RJ, Brazil

[3] Department of Bioorganic Chemistry, Leibniz Institute of Plant Biochemistry (IPB), Weinberg 3, 06114 Halle, Germany; wolfgang.brandt@ipb-halle.de (W.B.); aporzel@ipb-halle.de (A.P.)

* Correspondence: ivana@pharma.ufrj.br or ivafarma@gmail.com (I.C.R.L.); wessjohann@ipb-halle.de (L.A.W.); Tel.: +55-21-3938-6422 (I.C.R.L.); +49-345-5582-1300 (L.A.W.)

Content

Table S1. NMR data of compounds **1a** (major rotamer) and **1b** (minor rotamer) (600/150 MHz, solvent CD_3OD, +25 °C) S4
Scheme S1. Diagnostic ions from proposed fragmentation pathway in negative mode of ionization. RDA: Retro Diels Alder. QM: Quinone-methide. HR: Heterocyclic ring. S6
Scheme S2. Mass spectra MS and MS^2 of compound **1** highlighting the main fragments of proposed fragmentation pathway of proantocyanidin S7
Figure S1. ^{13}C NMR spectrum of compound **1** (150 MHz, CD_3OD) S8
Figure S2. ^{1}H NMR spectrum of compound **1** (600 MHz, CD_3OD) S9
Figure S3. ^{1}H,^{13}C HSQC NMR spectrum of compound **1** (600 MHz, CD_3OD) S10
Figure S4. ^{1}H,^{13}C HMBC NMR spectrum of compound **1** (600 MHz, CD_3OD) S11
Figure S5. ^{1}H,^{1}H DQFCOSY NMR spectrum of compound **1** (600 MHz, CD_3OD) S12
Figure S6. ^{1}H,^{1}H zTOCSY NMR spectrum of compound **1** (600 MHz, CD_3OD) S13
Figure S7. ^{1}H,^{1}H ROESY NMR spectrum of compound **1** (600 MHz, CD_3OD) S14
Figure S8. Calculated ECD spectrum (red curve) for the most stable conformation of the **C4-D8 *SSSS* M** atropisomer with a low similarity of 0.6071 and a shift of -30 nm to the experimental ECD spectrum (black curve), right) related structure with the dihedral angle (A10-C4-D8-D9) = -135.9°. S15
Figure S9. left) Calculated ECD spectrum (red curve) for the most stable conformation of the **C4-D8 *SSRR* P** atropisomer with a low similarity of 0.7511 and a shift of -26 nm to the experimental ECD spectrum (black curve). **right**) Related structure with the dihedral angle (A10-C4-D8-D9) = -131.9°. S15
Figure S10. left) Calculated ECD spectrum (red curve) for the most stable conformation of the **M** atropisomer with **C4-D8 *RRSS*** configuration with a low similarity of 0.6300 and a shift of 16 nm to the experimental ECD spectrum (black curve). **right**) Related structure with the dihedral angle (A10-C4-D8-D9) = 131.9°. S16
Figure S11. left) Calculated ECD spectrum (red curve) for the most stable conformation of the **M** atropisomer with **C4-D6 *RRRR*** configuration with a low similarity of 0.6709 and a shift of -5 nm to the experimental ECD spectrum (black curve). **right**) Related structure with the dihedral angle (A10-C4-D6-D5) = 133.5°. S17
Figure S12. left) Calculated ECD spectrum (red curve) for the most stable conformation of the **M** atropisomer with **C4-D6 *SSSS*** configuration with a similarity of 0.8379 and a shift of -1 nm to the experimental ECD spectrum (black curve), **right**) related structure with the dihedral angle (A10-C4-D6-D5) = 61.4° S18
Figure S13. left) Calculated ECD spectrum (red curve) for the most stable conformation of the **P** atropisomer **C4-D6 *SSRR*** isomer with a low similarity of 0.7730 and a shift of -30 nm to the experimental ECD spectrum (black curve), **right**) related structure with the dihedral angle (A10-C4-D6-D5) = -135.1°. S19
Figure S14. left) Calculated ECD spectrum (red curve) for the most stable conformation of the **P** atropisomer **C4-D6 *RRSS*** isomer with a similarity of 0.7863 and a shift of 27 nm to the experimental ECD spectrum (black curve), **right**) related structure with the dihedral angle (A10-C4-D6-D5) = -59.8°. S20
Figure S15. left) Calculated ECD-spectrum (red curve) for the **P** atropisomer with a relative energy of 2.9 kcal/mol of the **C4-D8 *SSSS*** isomer (M isomer: see Figure S12) with a similarity of 0.8631 with a shift of -3 nm to the experimental ECD-spectrum (black curve), **right**) related structure with the dihedral angle (A10-C4-D8-D9) = 60.6°. S21

Figure S16. left) Calculated ECD-spectrum (red curve) for the **M** atropisomer with a relative energy of 2.4 kcal/mol of the **C4-D8 *SSRR*** isomer (P isomer: see Figure 2B) with a similarity of 0.7720 with a shift of 20 nm to the experimental ECD-spectrum (black curve), **right**) related structure with the dihedral angle (A10-C4-D8-D9) = 61.2°. S22
Figure S17. left) Calculated ECD-spectrum (red curve) for the **P** atropisomer with a relative energy of 2.4 kcal/mol of the **C4-D8 *RRSS*** isomer (M isomer: see Figure S10) with a similarity of 0.7557 with a shift of -21 nm to the experimental ECD-spectrum (black curve), **right**) related structure with the dihedral angle (A10-C4-D8-D9) = -61.2°. S23
Figure S18. left) Calculated ECD-spectrum (red curve) for the **P** with a relative energy of 1.9 kcal/mol of the **C4-D6 *SSSS*** isomer atropisomer (M isomer: see Figure S12) with a similarity of 0.7564 and a shift of -26 nm to the experimental ECD-spectrum (black curve), **right**) related structure with the dihedral angle (A10-C4-D6-D5) = 133.5°. S24
Figure S19. left) Calculated ECD-spectrum (red curve) for the **M** atropisomer with a relative energy of 1.3 kcal/mol of the **C4-D6 *SSRR*** isomer (P isomer: see Figure 13) with a similarity of 0.8580 and a shift of -2 nm to the experimental ECD-spectrum (black curve), **right**) related structure with the dihedral angle (A10-C4-D6-D5) = 59.8°. S25
Figure S20. left) Calculated ECD-spectrum (red curve) for the **M** atropisomer with a relative energy of 0.86 kcal/mol of the **C4-D6 *RRSS*** isomer (P isomer: see Figure S14) with a similarity of 0.8427 and a shift of 29 nm to the experimental ECD-spectrum (black curve), **right**) related structure with the dihedral angle (A10-C4-D6-D5) = 134.2°. S26
Figure S21. Comparison of the calculated (red curve) with the experimental UV spectra for the **C4-D8 *RRRR* P** atropisomer with a similarity factor of 0.75. .... S27

Table S1. NMR data of compounds **1a** (major rotamer) and **1b** (minor rotamer) (600/150 MHz, solvent CD_3OD, +25 °C)

Pos.[a]	**1a** (major rotamer)		**1b** (minor rotamer)	
	δ ^{13}C [ppm]	δ ^{1}H [ppm] m (J [*Hz*])	δ ^{13}C [ppm]	δ ^{1}H [ppm] m (J [*Hz*])
C2	80.43	4.974 *dd* (11.6/2.0) ax	80.54	5.022 *dd* (11.6/1.8) ax
C3	36.71	2.640 *ddd* (13.2/12.0/11.6) ax; 1.836 *ddd* (13.2/5.8/2.0) eq	36.00	2.761 *ddd* (13.3/12.1/11.6) ax; 2.088 *ddd* (13.3/5.9/1.8) eq
C4	33.05	4.771 *ddd* (12.0/5.8/1.1) ax	32.89	4.899 *ddd* (12.1/5.9/1.0) ax
A5	129.84	6.709 *dd* (8.4/1.2)	129.66	6.594 *dd* (8.4/1.2)
A6	108.99	6.262 *dd* (8.4/2.5)	108.96	6.187 *dd* (8.4/2.5)
A7	156.75	---	156.78	---
A8	103.97	6.311 *d* (2.5)	103.80	6.223 *d* (2.5)
A9	157.26	---	157.30	---
A10	120.12	---	119.64	---
B1‘	134.77	---	134.56	---
B2‘/6‘	128.62	7.056 *d*-like (8.6)	128.80	7.308 *d*-like (8.6)
B3‘/5‘	115.95	6.660 *d*-like (8.6)	116.04	6.791 *d*-like (8.6)
B4‘	157.99	---	158.15	---
F2	79.30	4.731 br *s*-like ax	79.91	4.920 br *s*-like ax
F3	67.44	4.067 *ddd* (4.6/2.3/1.2) eq	67.13	4.270 *ddd* (4.7/3.0/1.5) eq
F4	29.35	2.846 *dd* (16.8/4.6) ax; 2.757 *ddd* (16.8/2.3/0.9) eq	29.69	2.960 *dd* (16.8/4.7) ax; 2.798 *ddd* (16.8/3.0/0.8) eq
D5	156.03	---	156.07[b]	---
D6	96.09	6.081 *s*	97.42	5.939 *s*
D7	155.74	---	155.96[b]	---
D8	110.05	---	110.14	---
D9	155.50	---	155.38	---
D10	100.78	---	99.63	---
E1‘	131.89	---	132.48	---
E2‘	114.14	6.540 *dd* (2.1/0.6)	115.22	7.014 *dd* (2.1/0.6)
E3‘	145.72	---	146.00	---
E4‘	145.43	---	145.75	---
E5‘	115.95	6.638 *d* (8.2)	115.99	6.768 *d* (8.2)
E6‘	119.76	6.132 *ddd* (8.2/2.1/0.6)	119.18	6.822 *dd* (8.2/2.1/0.6)

[a] numbering scheme see below (For the sake of clarity, the position number is preceded by the designation A, B, C, D, E, or F of the corresponding ring.); [b] interchangeable;

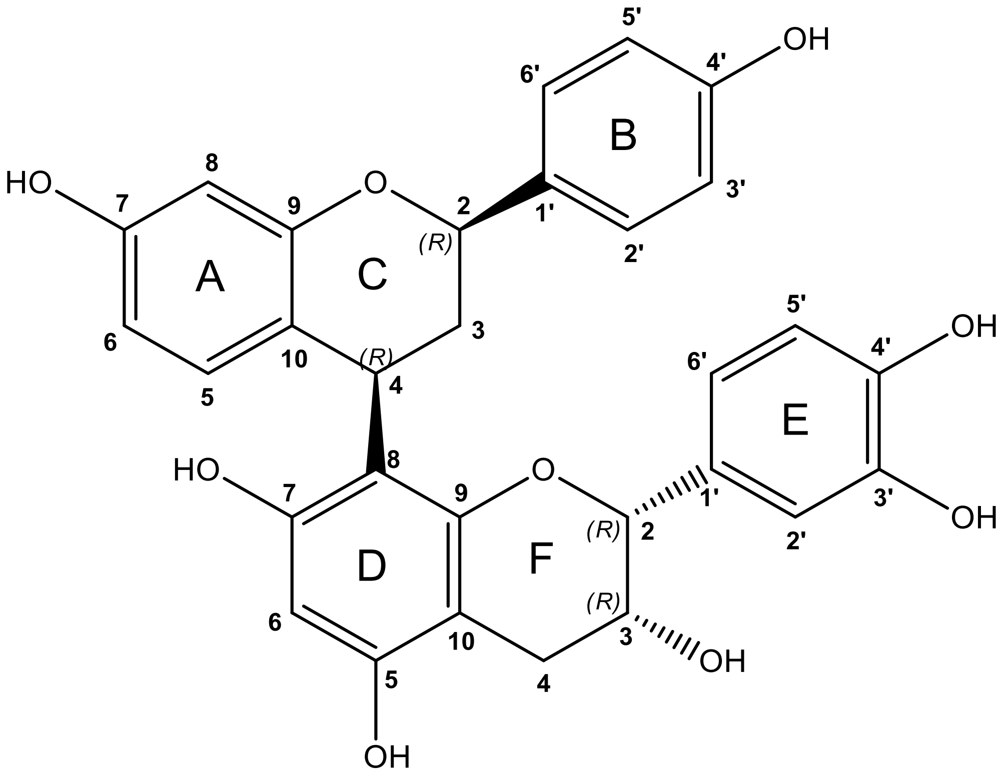
HO
OH
OH
OH
HO
OH
OH
A
B
C
D
E
F
(R)

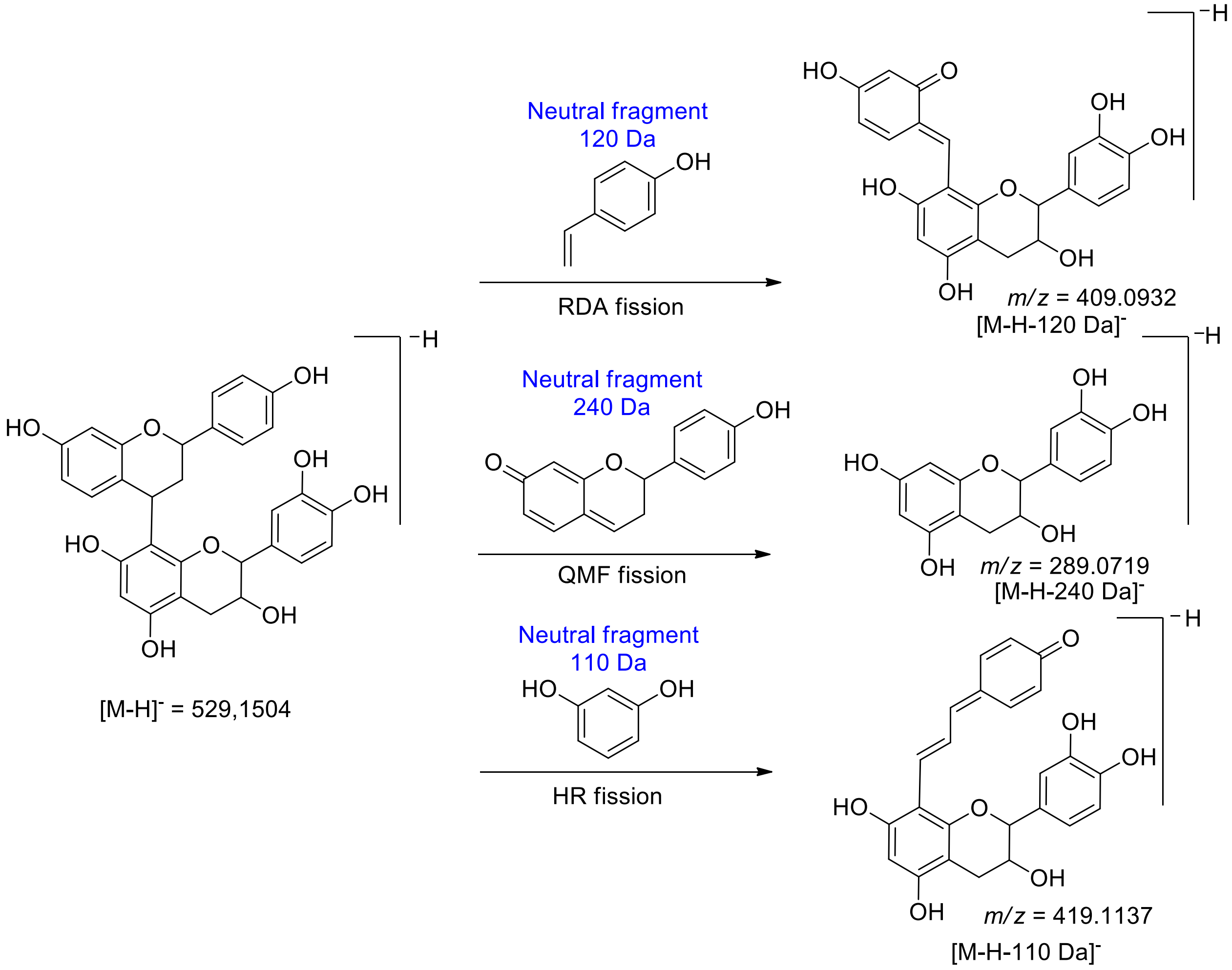

Scheme S1. Diagnostic ions from proposed fragmentation pathway in negative mode of ionization. RDA: Retro Diels Alder. QM: Quinone-methide. HR: Heterocyclic ring.

.

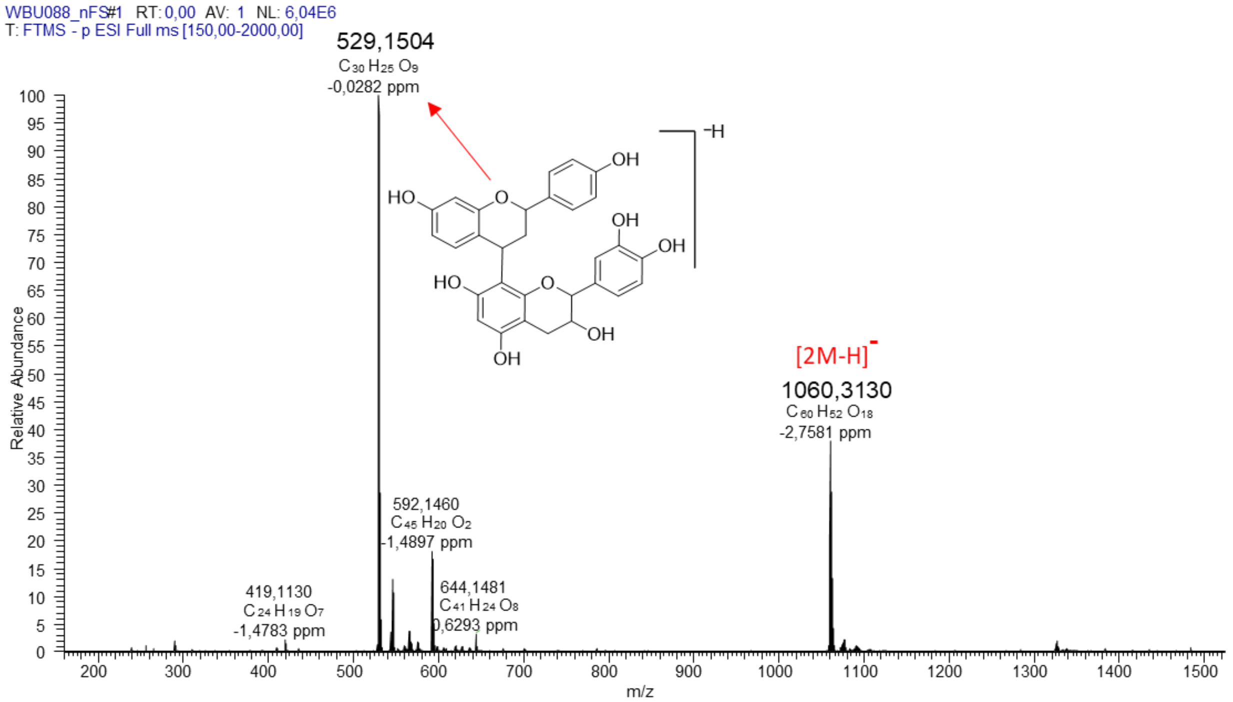

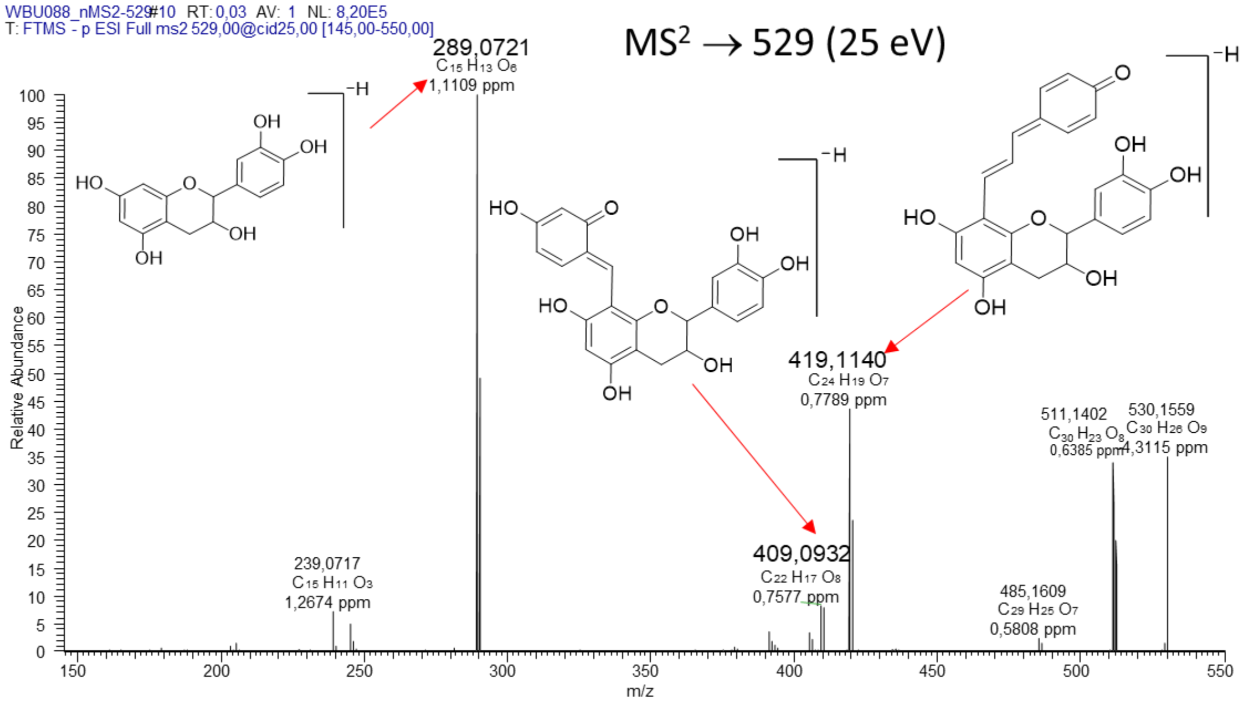

Scheme S2. Mass spectra MS and MS^2 of compound **1** highlighting the main fragments of proposed fragmentation pathway of proantocyanidin

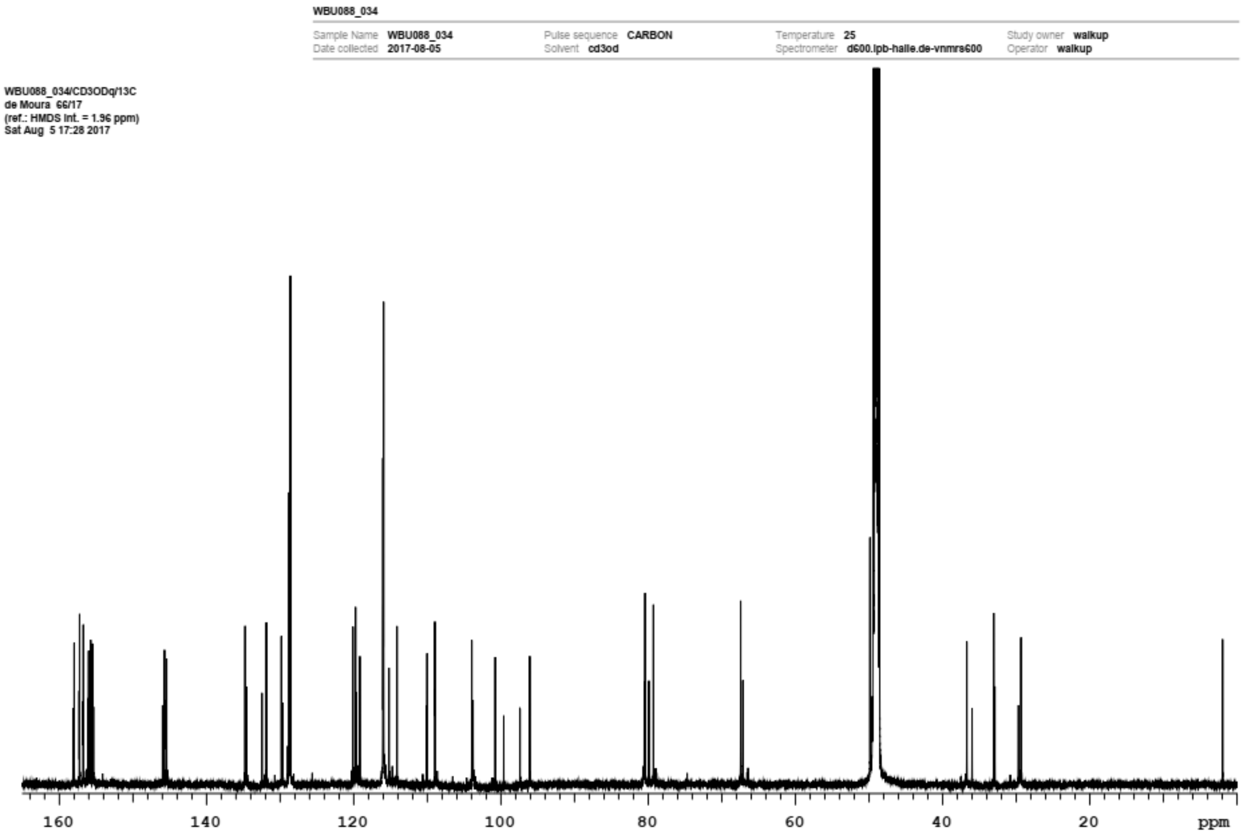

Figure S1. ^{13}C NMR spectrum of compound **1** (150 MHz, CD_3OD)

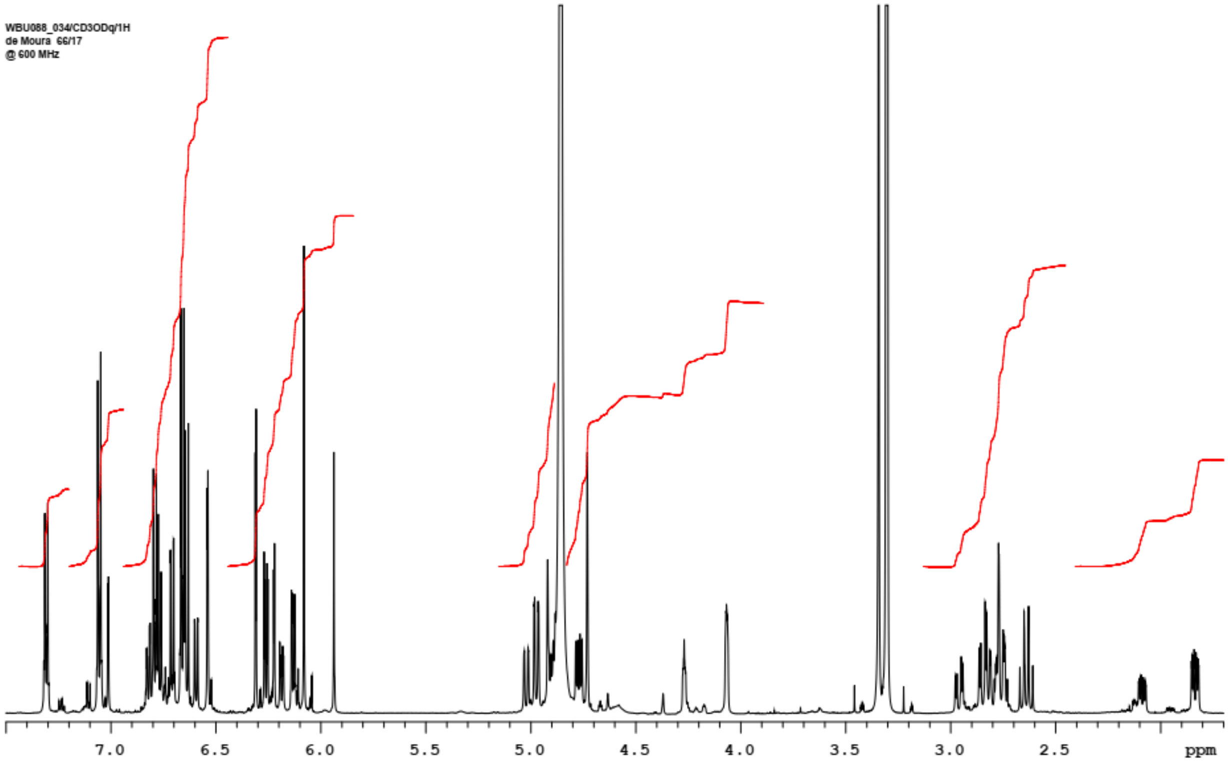

Figure S2. ^{1}H NMR spectrum of compound **1** (600 MHz, CD_3OD)

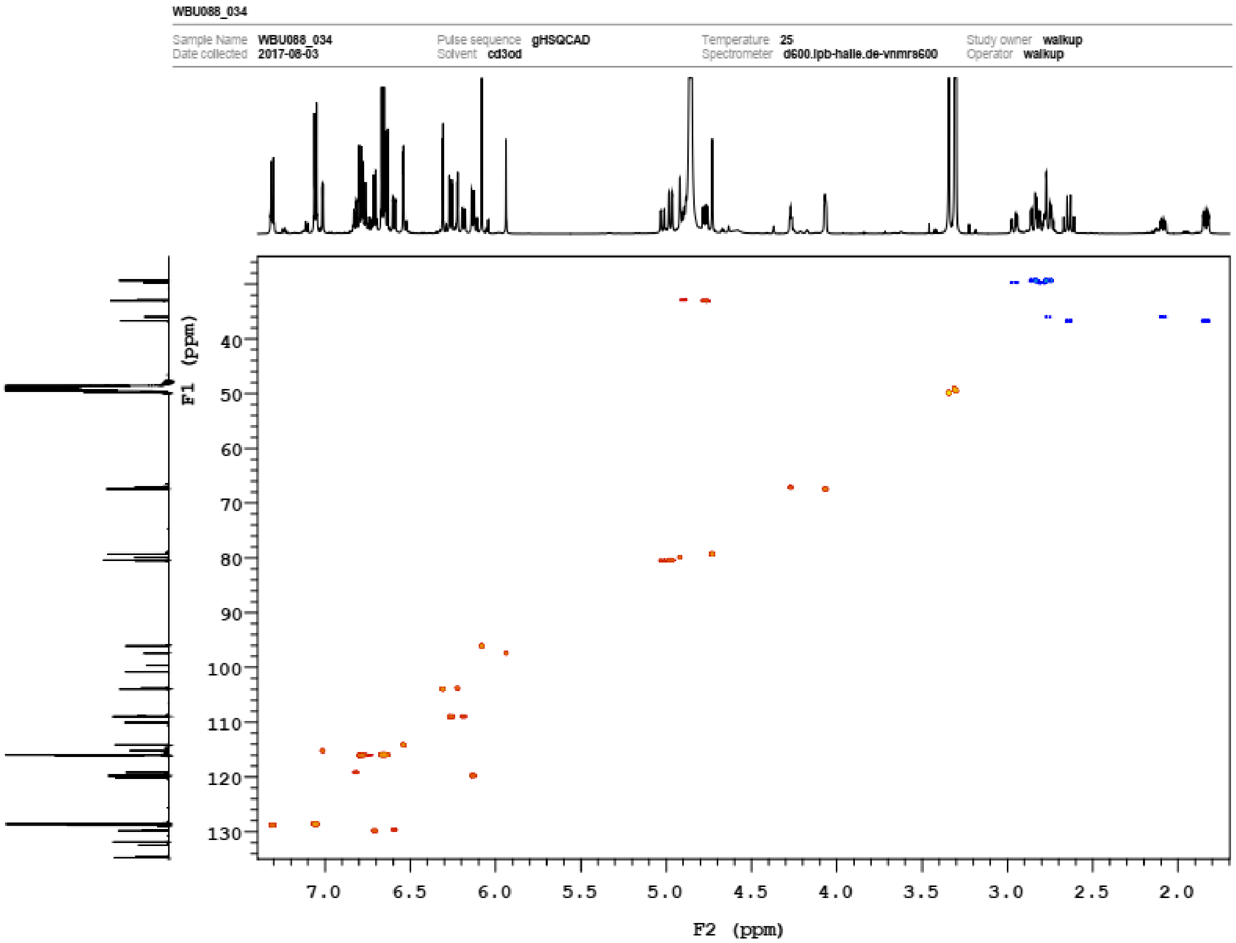

Figure S3. ^{1}H,^{13}C HSQC NMR spectrum of compound **1** (600 MHz, CD_3OD)

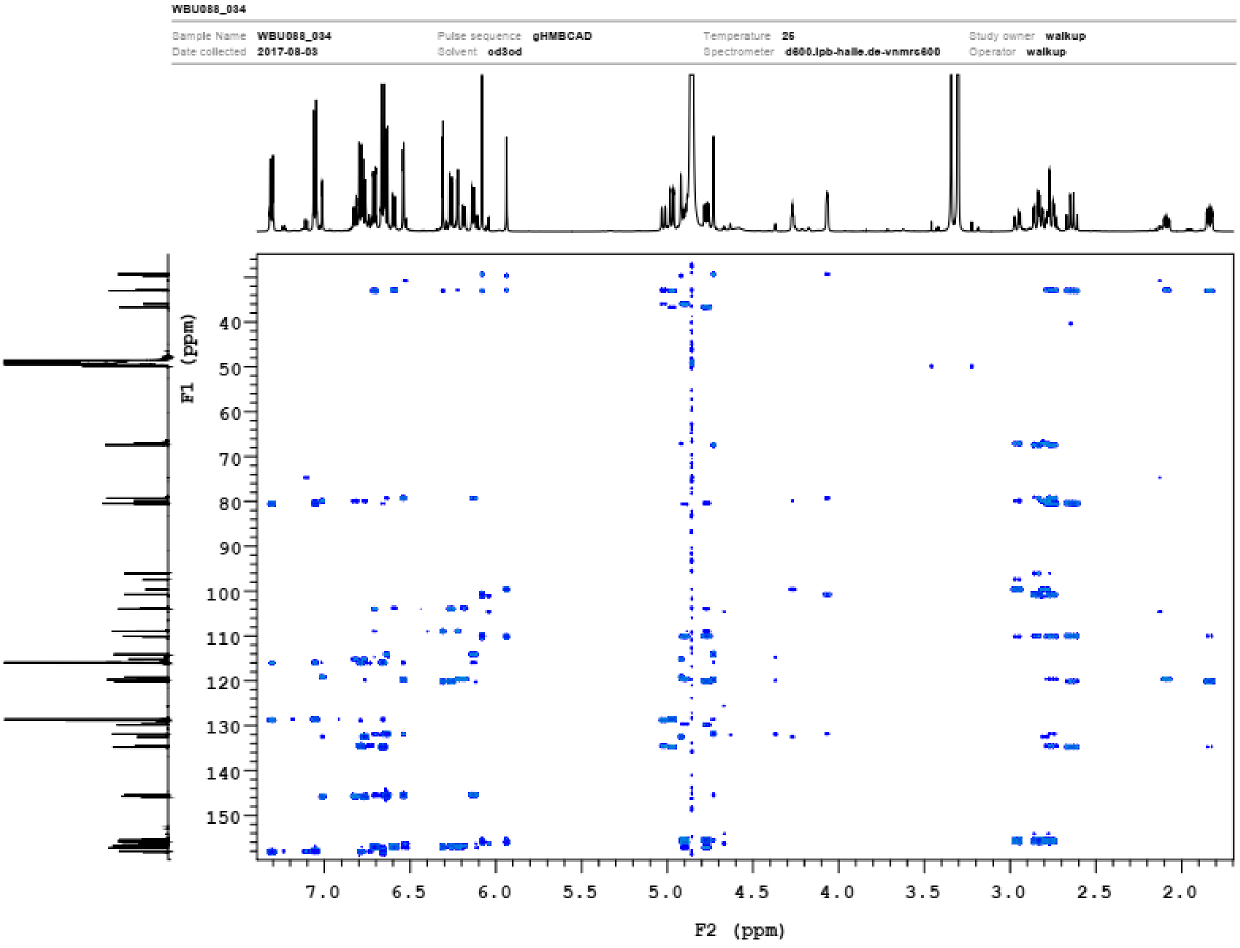

Figure S4. $^{1}H,^{13}C$ HMBC NMR spectrum of compound **1** (600 MHz, CD_3OD)

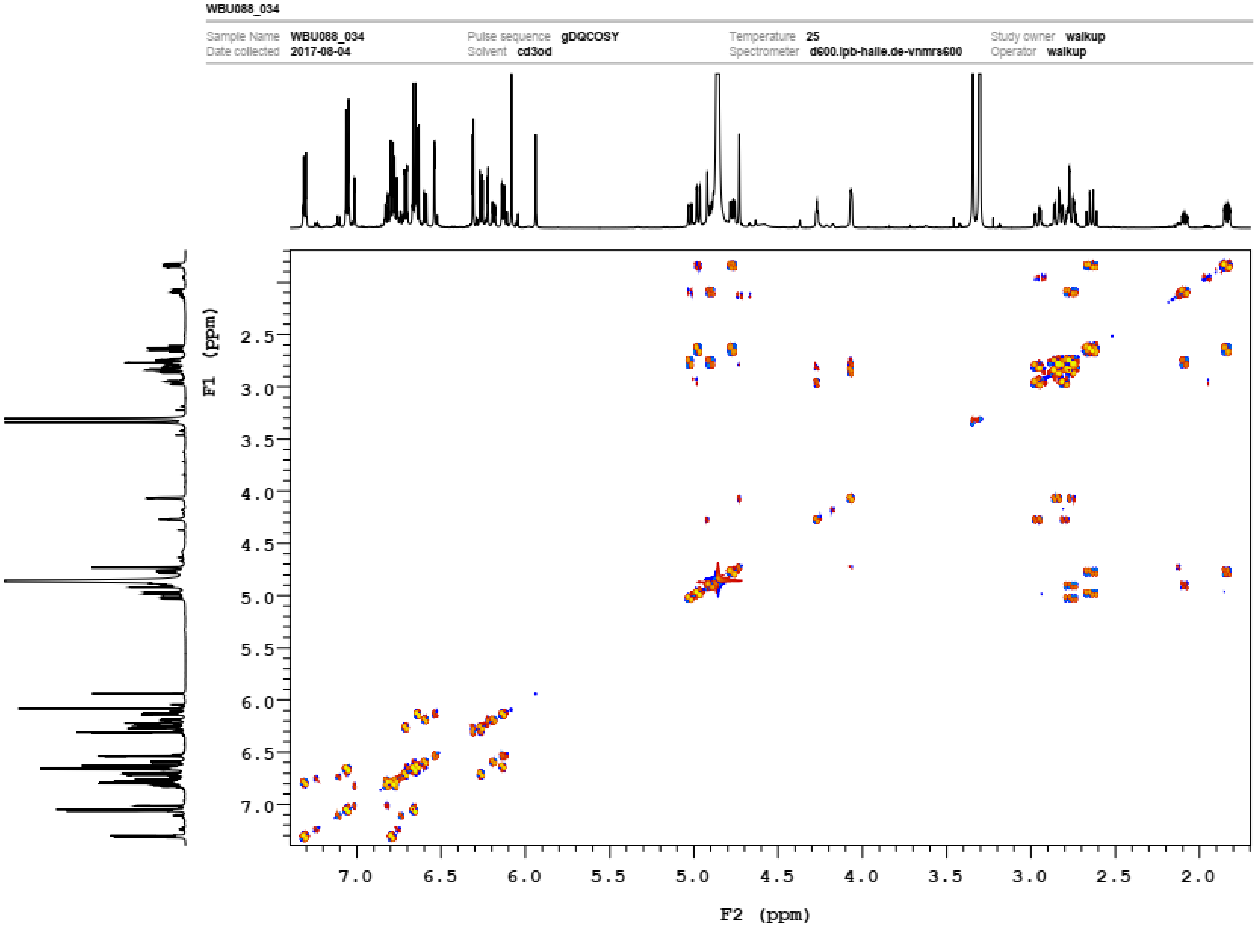

Figure S5. ^{1}H,^{1}H DQFCOSY NMR spectrum of compound **1** (600 MHz, CD_3OD)

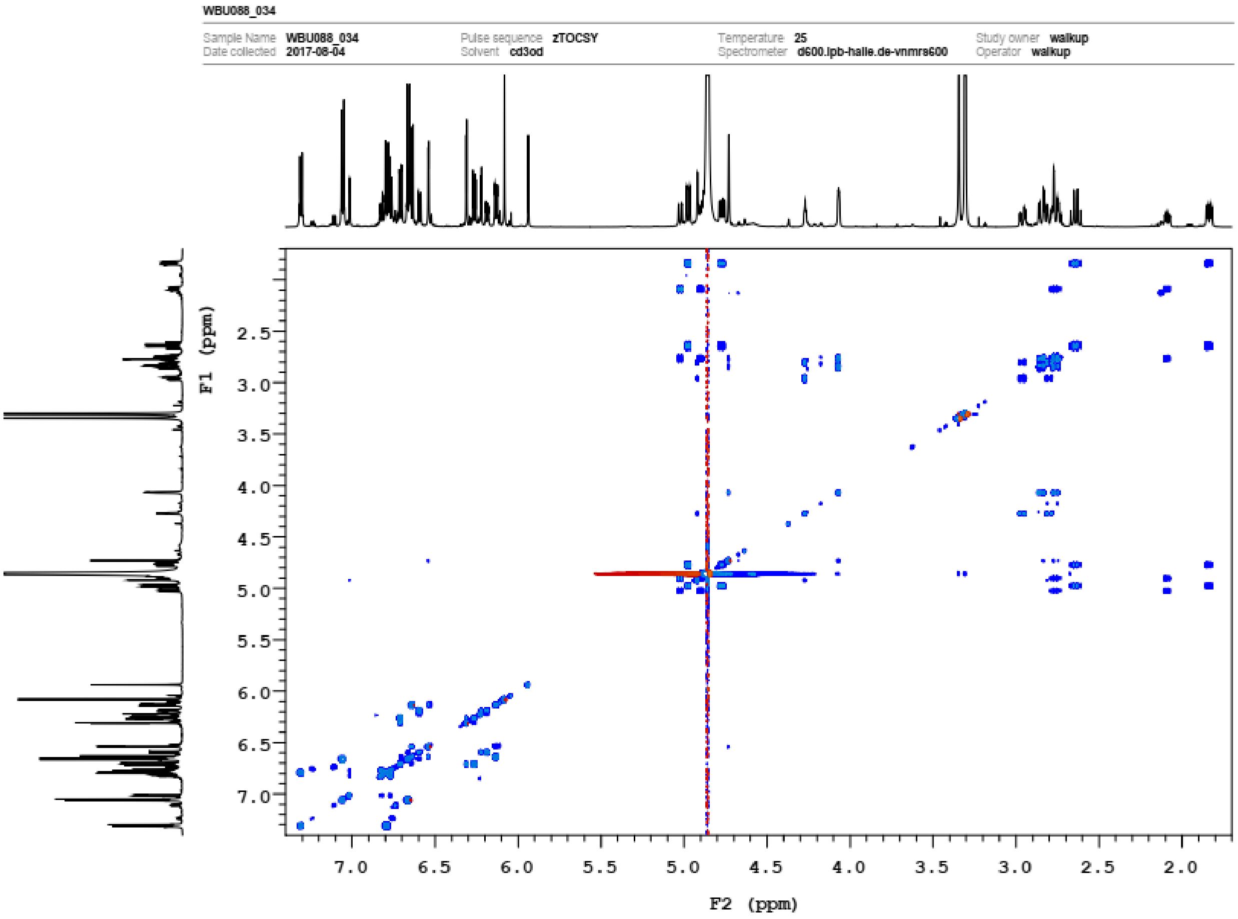

Figure S6. ^{1}H,^{1}H zTOCSY NMR spectrum of compound **1** (600 MHz, CD_3OD)

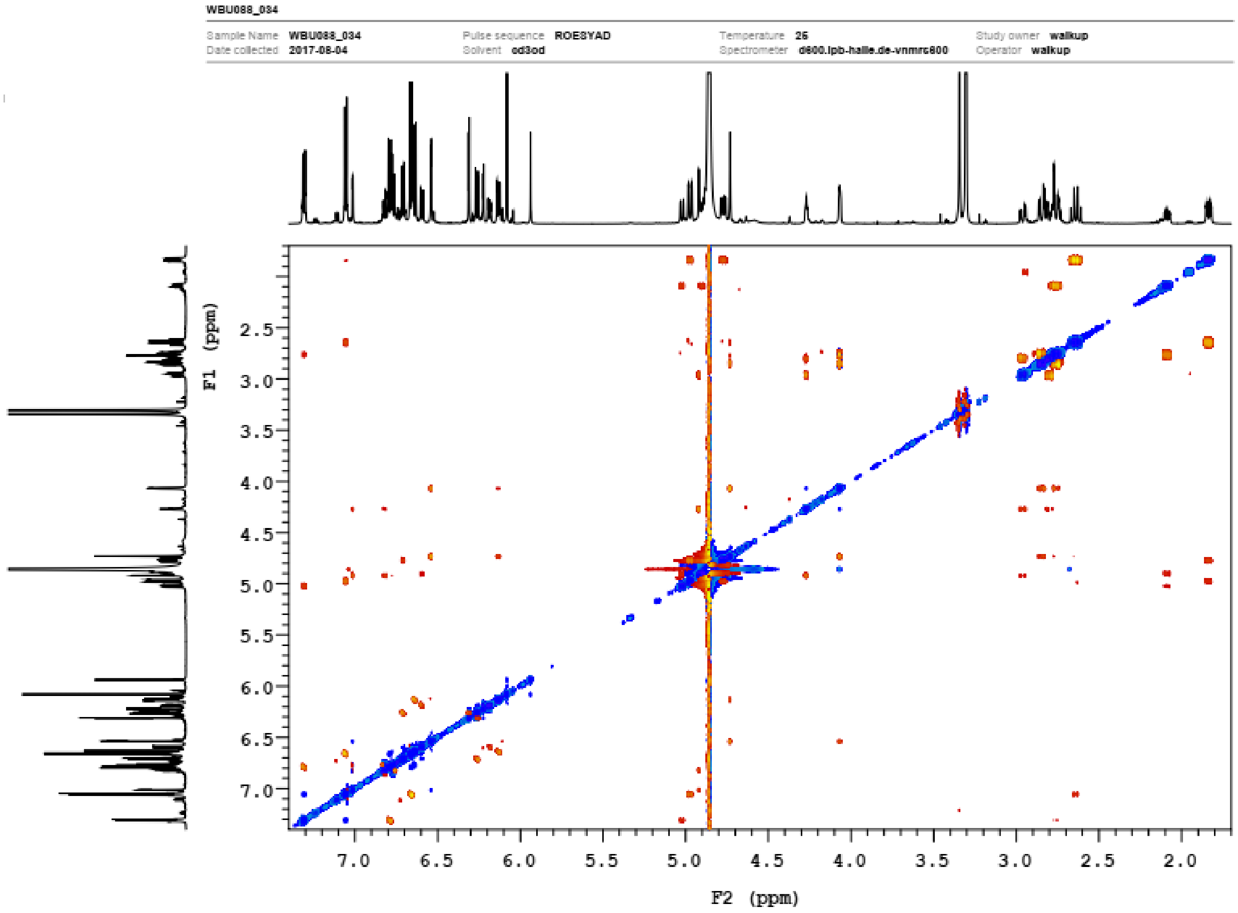

Figure S7. ^{1}H,^{1}H ROESY NMR spectrum of compound **1** (600 MHz, CD_3OD)

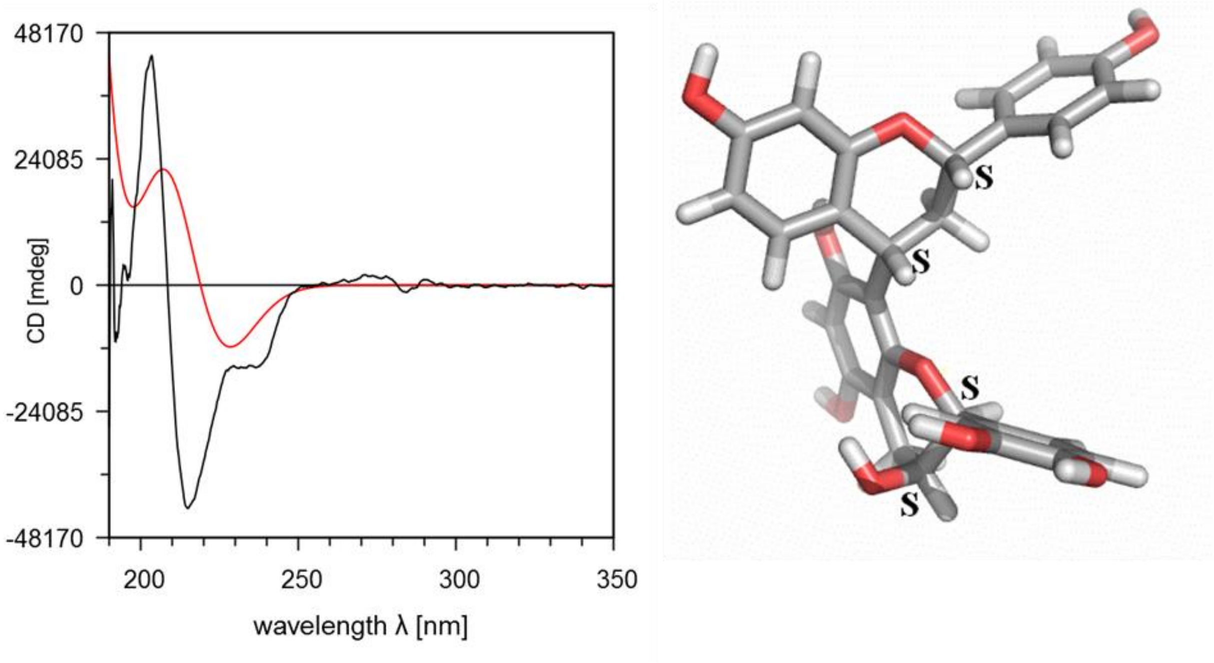

Figure S8. Calculated ECD spectrum (red curve) for the most stable conformation of the **C4-D8 SSSS M** atropisomer with a low similarity of 0.6071 and a shift of -30 nm to the experimental ECD spectrum (black curve), right) related structure with the dihedral angle (A10-C4-D8-D9) = -135.9°.

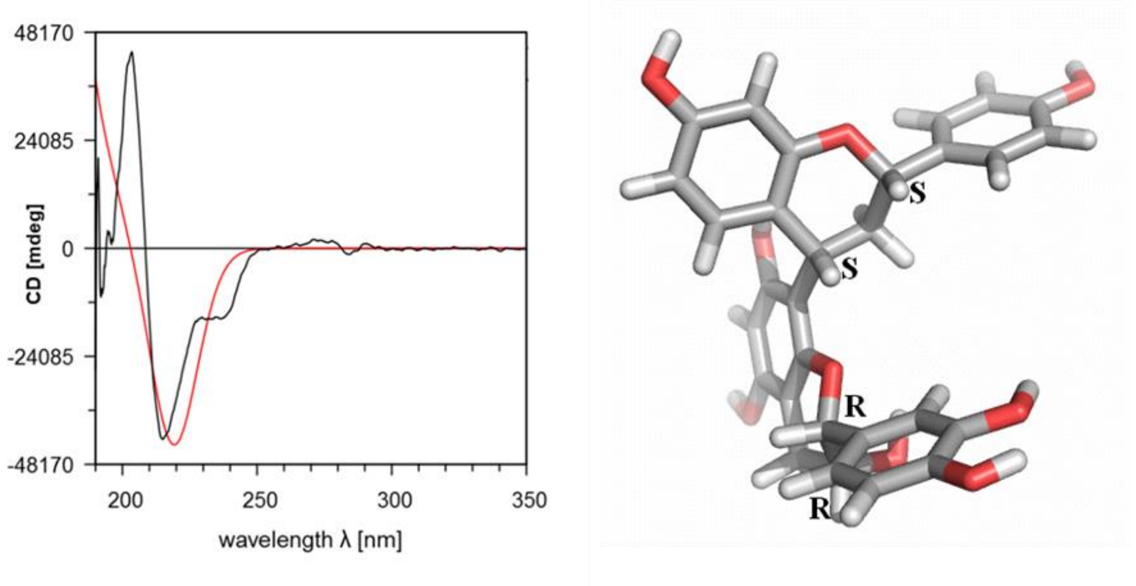

Figure S9. left) Calculated ECD spectrum (red curve) for the most stable conformation of the **C4-D8 *SSRR* P** atropisomer with a low similarity of 0.7511 and a shift of -26 nm to the experimental ECD spectrum (black curve). **right**) Related structure with the dihedral angle (A10-C4-D8-D9) = -131.9°.

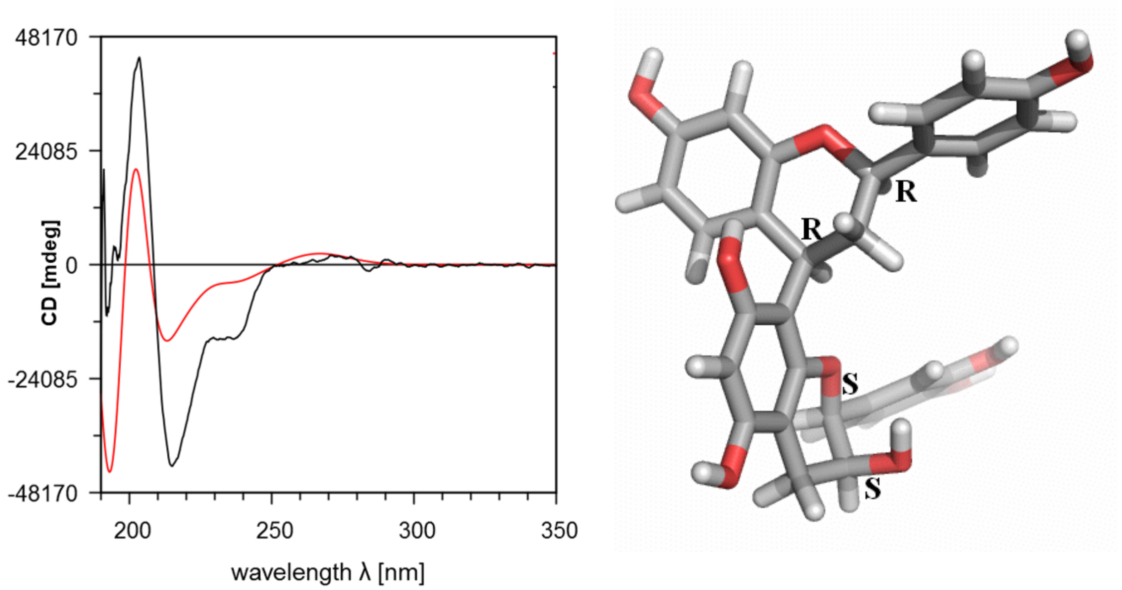

Figure S10. left) Calculated ECD spectrum (red curve) for the most stable conformation of the **M** atropisomer with **C4-D8** ***RRSS*** configuration with a low similarity of 0.6300 and a shift of 16 nm to the experimental ECD spectrum (black curve). **right**) Related structure with the dihedral angle (A10-C4-D8-D9) = 131.9°.

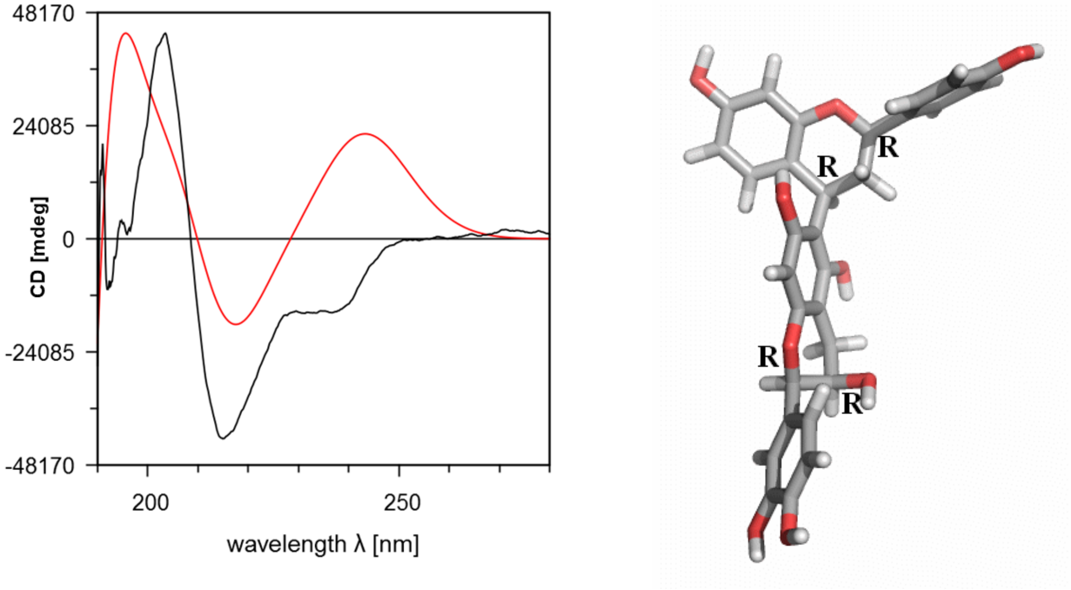

Figure S11. left) Calculated ECD spectrum (red curve) for the most stable conformation of the **M** atropisomer with **C4-D6** ***RRRR*** configuration with a low similarity of 0.6709 and a shift of -5 nm to the experimental ECD spectrum (black curve). **right**) Related structure with the dihedral angle (A10-C4-D6-D5) = 133.5°.

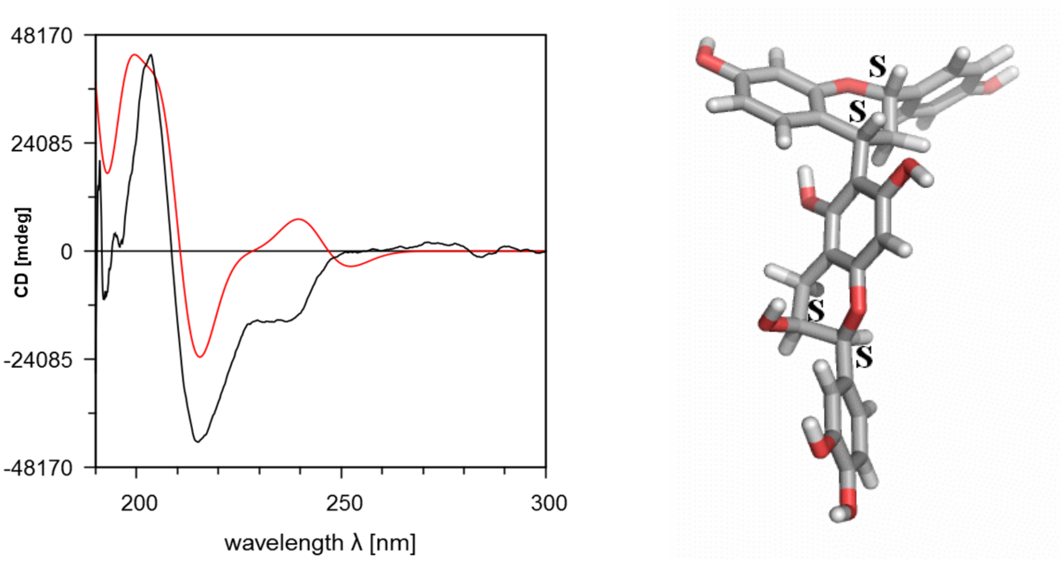

Figure S12. left) Calculated ECD spectrum (red curve) for the most stable conformation of the **M** atropisomer with **C4-D6** ***SSSS*** configuration with a similarity of 0.8379 and a shift of -1 nm to the experimental ECD spectrum (black curve), **right**) related structure with the dihedral angle (A10-C4-D6-D5) = 61.4°.

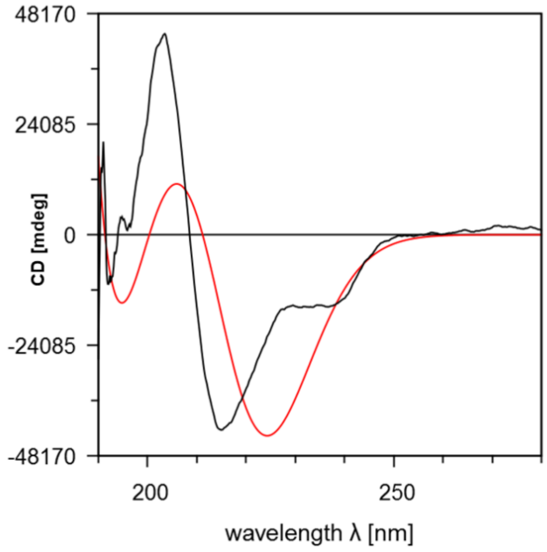

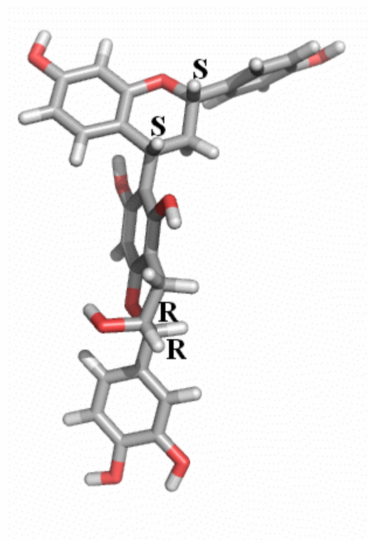

Figure S13. left) Calculated ECD spectrum (red curve) for the most stable conformation of the **P** atropisomer **C4-D6 *SSRR*** isomer with a low similarity of 0.7730 and a shift of -30 nm to the experimental ECD spectrum (black curve), **right**) related structure with the dihedral angle (A10-C4-D6-D5) = -135.1°.

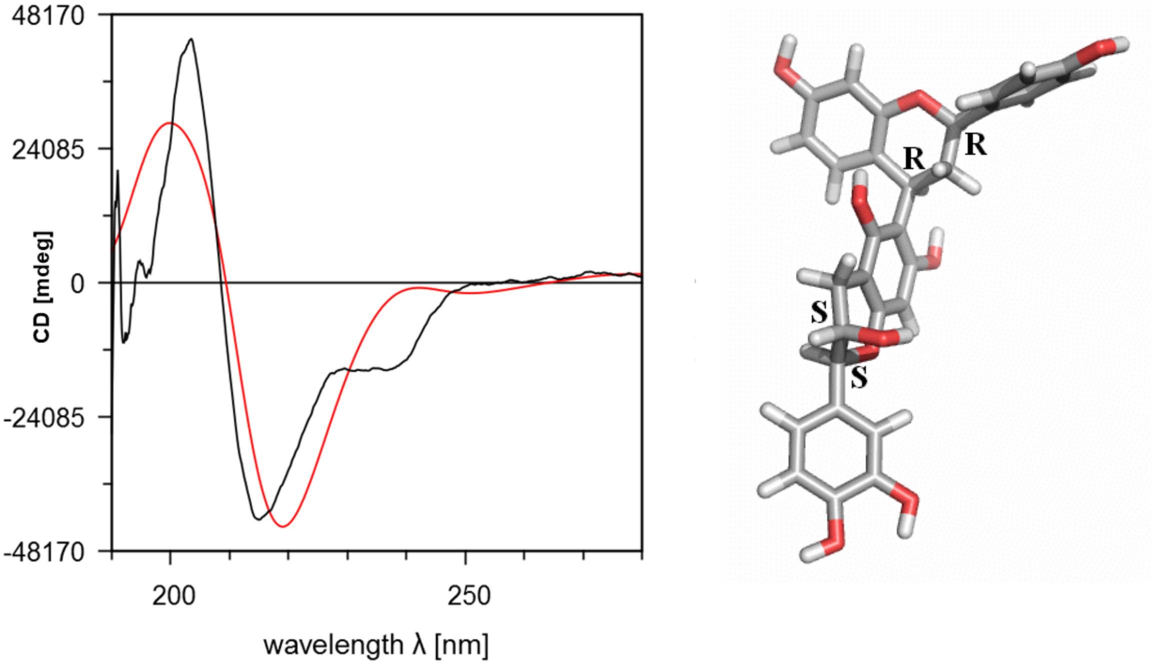

Figure S14. left) Calculated ECD spectrum (red curve) for the most stable conformation of the **P** atropisomer **C4-D6** ***RRSS*** isomer with a similarity of 0.7863 and a shift of 27 nm to the experimental ECD spectrum (black curve), **right**) related structure with the dihedral angle (A10-C4-D6-D5) = -59.8°.

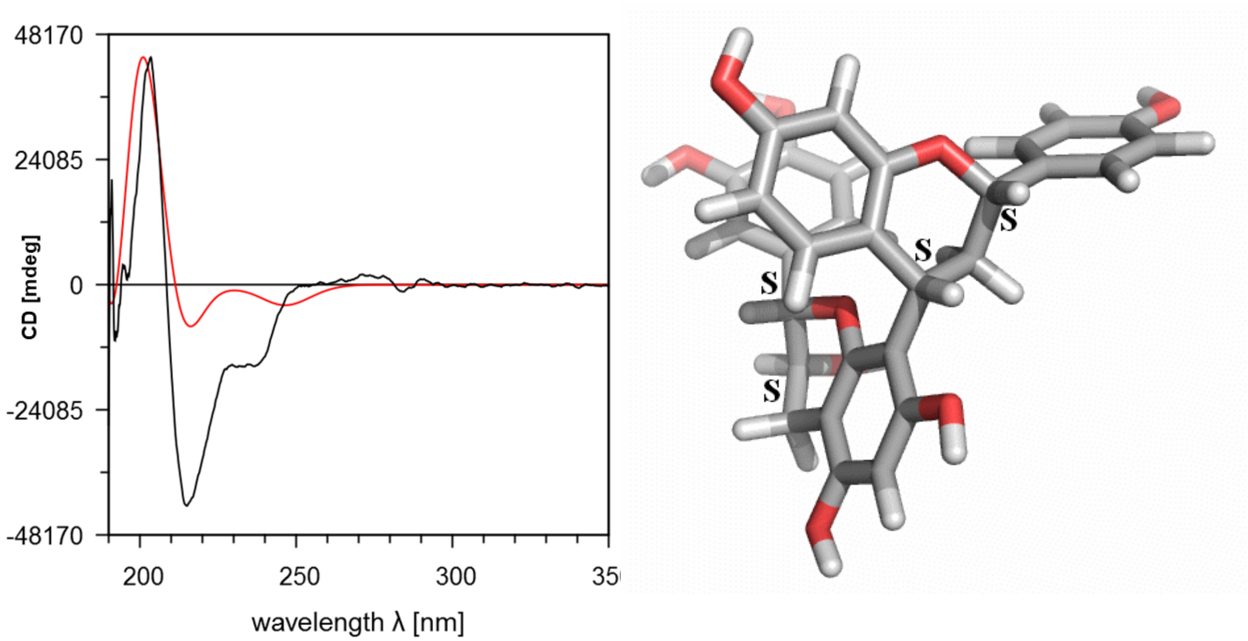

Figure S15. left) Calculated ECD-spectrum (red curve) for the **P** atropisomer with a relative energy of 2.9 kcal/mol of the **C4-D8** ***SSSS*** isomer (M isomer: see Figure S12) with a similarity of 0.8631 with a shift of -3 nm to the experimental ECD-spectrum (black curve), **right**) related structure with the dihedral angle (A10-C4-D8-D9) = 60.6°.

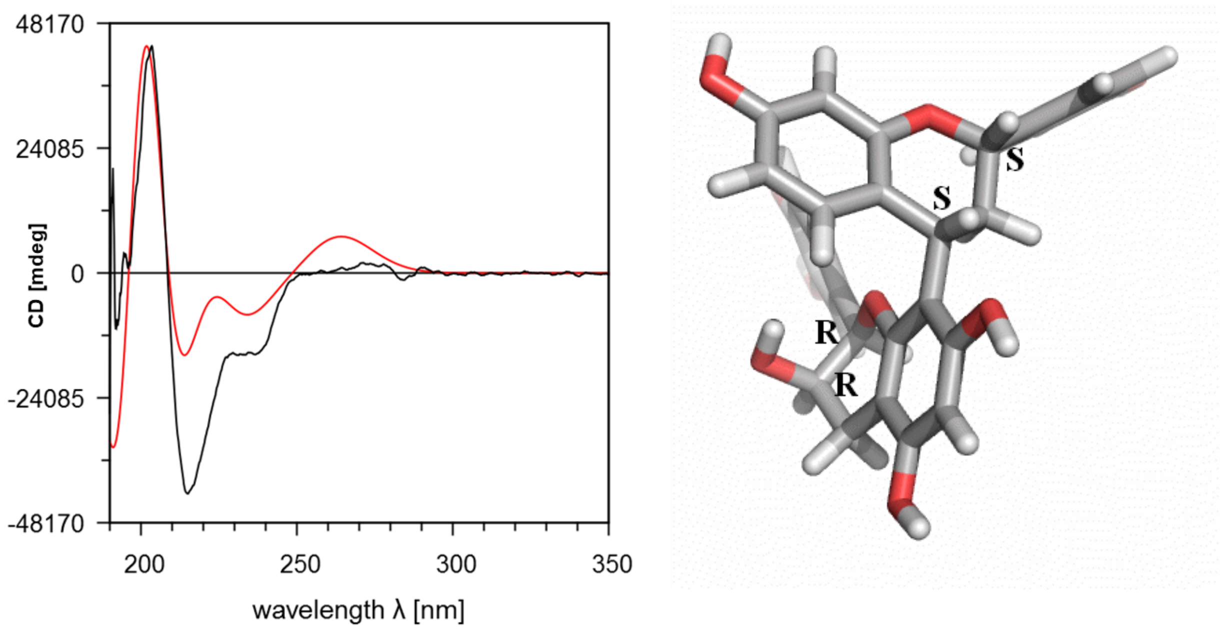

Figure S16. left) Calculated ECD-spectrum (red curve) for the **M** atropisomer with a relative energy of 2.4 kcal/mol of the **C4-D8** ***SSRR*** isomer (P isomer: see Figure S9) with a similarity of 0.7720 with a shift of 20 nm to the experimental ECD-spectrum (black curve), **right**) related structure with the dihedral angle (A10-C4-D8-D9) = 61.2°.

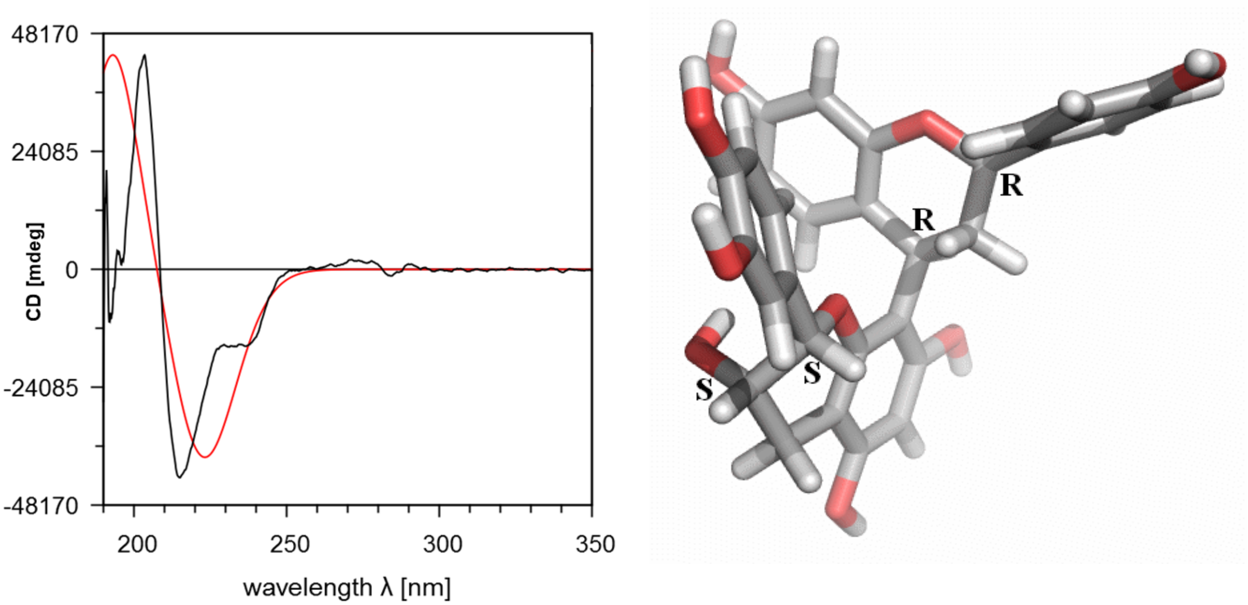

Figure S17. left) Calculated ECD-spectrum (red curve) for the **P** atropisomer with a relative energy of 2.4 kcal/mol of the **C4-D8 *RRSS*** isomer (M isomer: see Figure S10) with a similarity of 0.7557 with a shift of -21 nm to the experimental ECD-spectrum (black curve), **right**) related structure with the dihedral angle (A10-C4-D8-D9) = -61.2°.

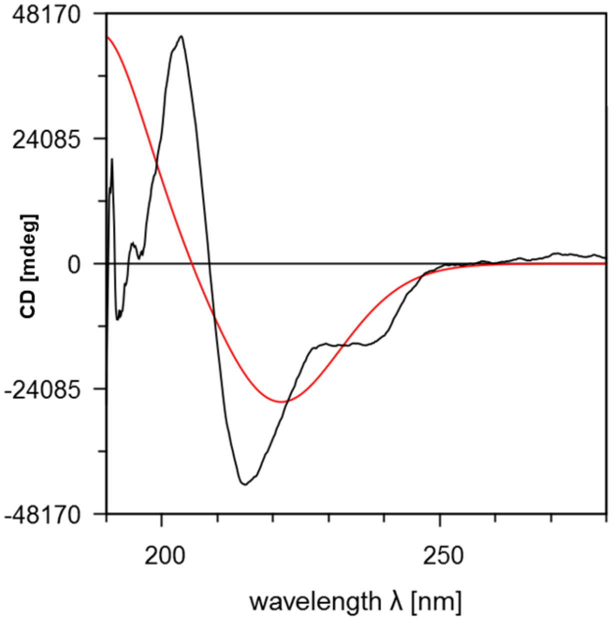

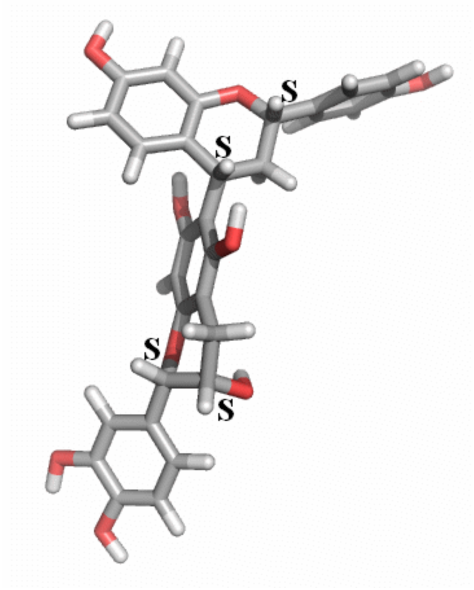

Figure S18. left) Calculated ECD-spectrum (red curve) for the **P** with a relative energy of 1.9 kcal/mol of the **C4-D6 *SSSS*** isomer atropisomer (M isomer: see Figure S12) with a similarity of 0.7564 and a shift of -26 nm to the experimental ECD-spectrum (black curve), **right**) related structure with the dihedral angle (A10-C4-D6-D5) = 133.5°.

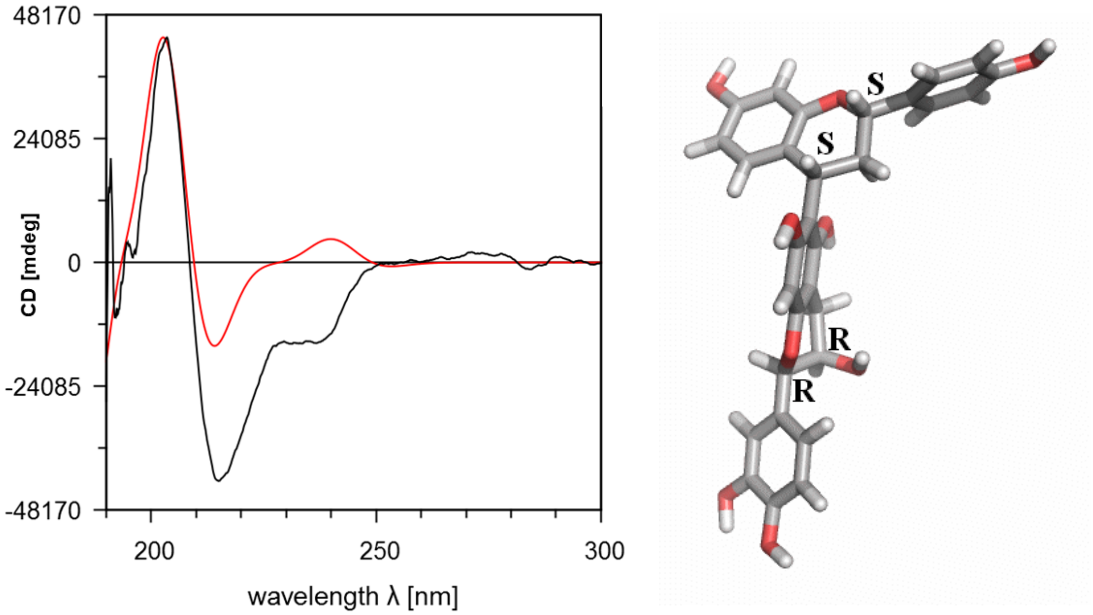

Figure S19. left) Calculated ECD-spectrum (red curve) for the **M** atropisomer with a relative energy of 1.3 kcal/mol of the **C4-D6** ***SSRR*** isomer (P isomer: see Figure 13) with a similarity of 0.8580 and a shift of -2 nm to the experimental ECD-spectrum (black curve), **right**) related structure with the dihedral angle (A10-C4-D6-D5) = 59.8°.

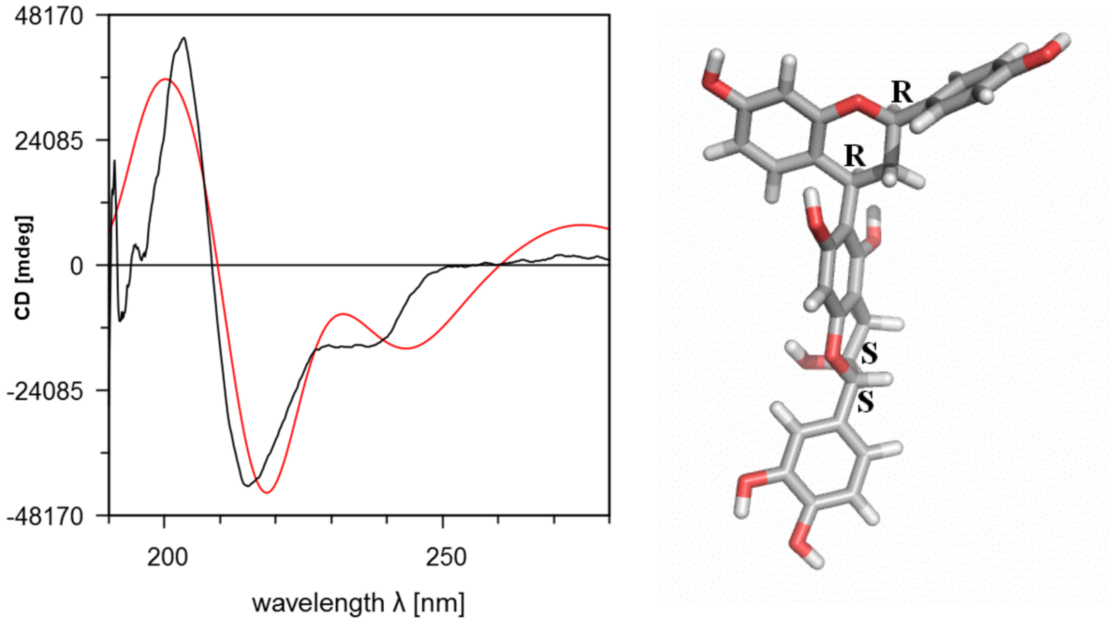

Figure S20. left) Calculated ECD-spectrum (red curve) for the **M** atropisomer with a relative energy of 0.86 kcal/mol of the **C4-D6** ***RRSS*** isomer (P isomer: see Figure S14) with a similarity of 0.8427 and a shift of 29 nm to the experimental ECD-spectrum (black curve), **right**) related structure with the dihedral angle (A10-C4-D6-D5) = 134.2°.

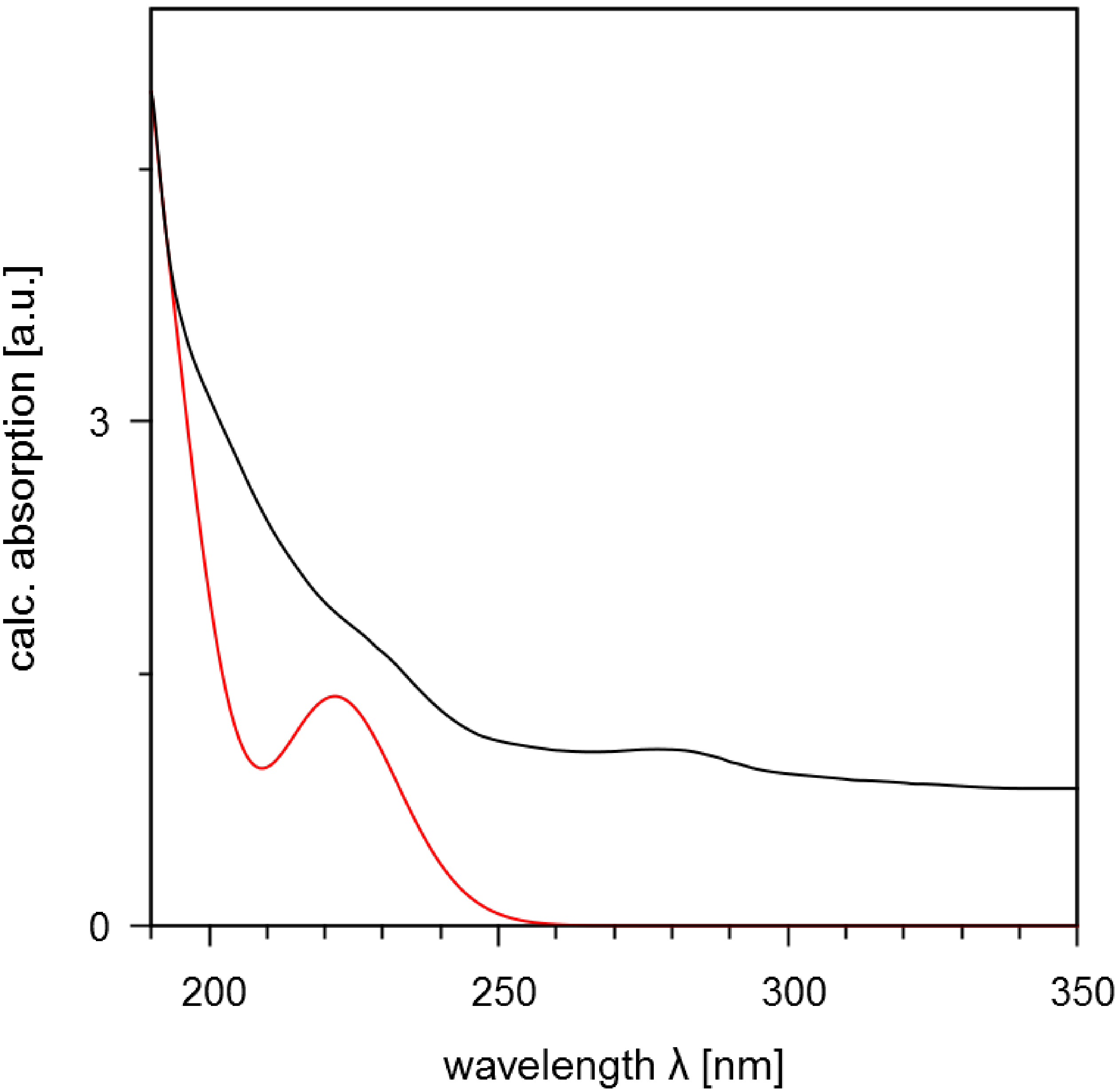

Figure S21. Comparison of the calculated (red curve) with the experimental UV spectra for the **C4-D8** ***RRRR*** **P** atropisomer with a similarity factor of 0.75.
